# Development and Evaluation of Stability of a Gel Formulation Containing the Monoterpene Borneol

**DOI:** 10.1155/2016/7394685

**Published:** 2016-05-09

**Authors:** Milla Gabriela Belarmino Dantas, Silvio Alan Gonçalves Bomfim Reis, Camila Mahara Dias Damasceno, Larissa Araújo Rolim, Pedro José Rolim-Neto, Ferdinando Oliveira Carvalho, Lucindo José Quintans-Junior, Jackson Roberto Guedes da Silva Almeida

**Affiliations:** ^1^Center for Studies and Research of Medicinal Plants, Federal University of Vale do São Francisco, 56.304-205 Petrolina, PE, Brazil; ^2^Federal University of Pernambuco, 50.670-901 Recife, PE, Brazil; ^3^Federal University of Vale do São Francisco, 56.304-205 Petrolina, PE, Brazil; ^4^Department of Physiology, Federal University of Sergipe, 49.100-000 São Cristóvão, SE, Brazil

## Abstract

Borneol is a bicyclic monoterpenoid alcohol commonly used in traditional Chinese and Indian medicine. It is extracted from the essential oil of various medicinal plants. It has antibacterial, analgesic, and anti-inflammatory action proven in studies that used oral and intraperitoneal applications of this monoterpene in mice. The current study was designed to develop a topical gel formulation containing the monoterpene borneol using carbopol as gel base and to evaluate its stability. The prepared formulation was subjected to physical characterization and physical-chemistry assessment. The gel was prepared from carbopol and 5% of borneol. The prepared gel was subjected to pharmacotechnical tests such as its pH, viscosity, conductivity, spreadability, centrifugation, and accelerated stability with freezing-thaw cycle. The borneol was successfully incorporated into the carbopol formulation. Borneol gel (BG5) showed good stability after eight months of its development and after 12 days in the freeze-thaw cycle, not showing statistical difference in pH value, conductivity, and viscosity before and after test. Furthermore, the formulation showed a good spreadability. Therefore, it was concluded that the formulation could be very promising alternative for the topical or transdermal treatment of skin diseases.

## 1. Introduction

The topical delivery of drugs is an attractive method for local and systemic treatments and is commonly used in the treatment of inflammatory conditions like dermatological diseases and musculoskeletal injuries [1–3]. Topical application has many advantages over the conventional dosage forms, especially to avoid some serious systemic adverse effects [4].

When the drug is delivered topically it can penetrate deeper into skin and hence give better absorption [2, 5]. Topical preparation prevents the metabolism of drug in the liver, avoids gastrointestinal disorders and the risks and inconveniences of intravenous therapy, and avoids the risks associated with the varied conditions of absorption, like pH changes, presence of enzymes, and gastric emptying time. Furthermore, the bioavailability of the drug is increased and its action occurs directly at the action site [6, 7].

A wide variety of pharmaceutical dosage forms can be used in the delivery system for topical drugs. The most used ones are gels, creams, and ointments, followed by sprays and liquid preparations [8, 9]. The topical delivery with gels can increase the resistance time of the drug on the skin and improve the delivery and release of the substance by increasing the residence time at the injection site [10]. Furthermore, transdermal delivery of some drugs such as nonsteroidal anti-inflammatory drugs (NSAIDs) using gel is proven effective for a variety of clinical conditions [11].

Borneol (C_10_H_18_O) is a bicyclic monoterpenoid alcohol commonly used in traditional Chinese and Indian medicine and is found in more than 60 products based on medicinal plants [12–14]. The borneol is extracted from the essential oil of various medicinal plants and has the ability to accelerate the opening of the blood-brain barrier and increase the bioavailability of drugs in the brain tissue [15, 16]. It has antibacterial, analgesic, and anti-inflammatory action proven in studies that used oral and intraperitoneal applications of this monoterpene in mice [17, 18].

Although the easy borneol penetration into the nervous tissue is reported and it is a potent analgesic and anti-inflammatory, there are no reports in the literature about borneol penetration of the skin and its topical or transdermal effect. Therefore, the aim of this study was to develop an effective, stable topical gel containing borneol 5% (BG5).

## 2. Materials and Methods

### 2.1. Materials

The borneol was purchased from Sigma-Aldrich®, and carbopol 940, EDTA, methylparaben, and triethanolamine were purchased from Via Farma®.

### 2.2. Preparation of Borneol Gel

The exact amount of carbopol 940 (1%), propylene glycol (7%), and methylparaben (0.1%) was dispersed in distilled water. The carbopol dispersion was kept at rest for 24 hours to allow for the complete swelling. Then the blended carbopol was mixed with continuous stirring, ultrasound, and hot plate to form gel aspect. Dispersion obtained was neutralized with required quantity of triethanolamine to obtain pH 5.0 to 5.5. The carbopol remained in a plastic jar for a week at room temperature. Then, a concentration corresponding to 5% of borneol was diluted with propylene glycol and added to the carbopol. The BG5 was stored in a glass jar and kept at room temperature for 8 months for physical and chemistry analysis.

### 2.3. Macroscopic Analysis of Formulation

The prepared BG5 formulation was inspected visually for its color, homogeneity, consistency, and spreadability. The clarity was determined by using the natural light and all the macroscopic analyses were realized comparing with carbopol.

### 2.4. Accelerated Stability and Physicochemical Analysis of Borneol Gel

The BG5 was submitted to accelerated stability tests. The gel was kept at room temperature (20–25°C) for evaluation to color, odor, pH, viscosity, and conductivity. For analysis of the resistance to freeze-thaw cycle, the BG5 was kept at a temperature of 4°C and 40°C for 12 days. The tests were performed eight months after the production of the formulation. Each test was done in triplicate with samples of 30 grams each.

### 2.5. Freeze-Thaw Cycle

The BG5 sample was subjected to freeze and thaw cycle; the test was performed in 12 days with six cycles. In each cycle, the substance remained in a particular temperature for a period of 24 hours. The temperature in the refrigerator was 5 ± 2°C and 40°C in the greenhouse.

### 2.6. Centrifugation Test

To perform the centrifugation test, 10 g of formulation was added in a tapered test tube. In centrifugation, the sample gel was subjected to a cycle of 3000 rpm for 30 minutes at room temperature. Centrifugation was performed with Model Centribio® 80-2B equipment.

### 2.7. Spreadability

The spreadability of the BG5 was measured by spreading of 0.5 g of the gel on a circle of 2 cm diameter premarked on a glass plate and then a second glass plate was employed. Half kilogram of weight was permitted to rest on the upper glass plate for 5 min [19, 20]. The diameter of the circle after spreading of the gel was determined.

### 2.8. Conductivity and pH Analysis

The pH and conductivity of BG5 formulation were determined by using digital pH meter (MSTecnopon equip. Special LTDA). The glass electrode was calibrated with the solutions determined for the equipment (pH of 4.00 and 7.00), and the conductivity measurement was done in millivolts (mV). The preparation was left for about 15 min for attaining equilibrium while measuring. The analysis of pH and conductivity of formulation were done in triplicate and average values were calculated.

### 2.9. Viscosity Study

The viscosity measurement of the borneol gel was performed with a Viscometer (Quimis® MOD 0860M21). The gel was rotated at 10, 20, 30, 40, 50, and 60 rotations per minute. At each speed, the corresponding dial reading was noted.

### 2.10. Statistical Analysis

The BG5 formulation was tested in triplicate, and each analysis was duplicated. Effects of formulation variables after freeze-thaw cycle were tested for significance by using Student's *t*-test using Graph Pad Prism software 5.0 version (Graph Pad Software Inc., San Diego, CA, USA) and the *p* values < 0.05 were considered.

## 3. Results and Discussion

The borneol gel formulation (BG5) was assessed for its macroscopic characteristics and qualities such as color, aspect, and aroma. The borneol gel formulation has a smooth texture and white color transparent and homogeneous and characteristic odor of borneol extract. The BG5 characteristics remained similar eight months after development and there was no difference in aspect of BG5 before and after freeze-thaw cycle.

The pH values of the BG5 were found to be in the range from 3.95 ± 0.01 to 3.83 ± 0.03, which was expected since the carbopol was formulated with pH between 5 and 5.5 because these are values sufficient to obtain a good viscosity and clarity of the gel [21]. Furthermore, the borneol is also considered acidic [22]. This pH value showed that the BG5 probably would not produce skin irritation. The conductivity values of BG5 also remained stable; statistical difference in pH values and conductivity before and after freezing-thaw cycle was not verified. Hence, the prepared borneol gel is suitable for topical application.  Table 1 shows the physical and chemical values of BG5 before and after twelve days of the freeze and thaw cycle.

The spreadability of BG5 was considered high by having a low spread of time. The therapeutic efficacy of gels depends on their spread. The gel spreading helps in the uniform application of the gel to the skin, so the prepared gels must have a good spreadability and satisfy the ideal quality in topical application. Furthermore, this is considered an important factor in patient compliance with treatment.

**Figure 1:**
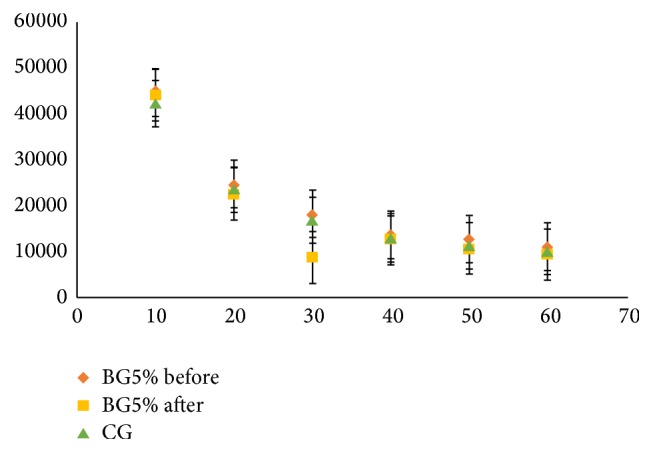
Effect of shear rate on the viscosity of borneol gel 5% (BG5%) before and after the freeze-thaw cycle and carbopol® (CG) gel formulation. Values are mean ± standard error.

The consistency of the substance is one of the most important features to analgesic and anti-inflammatory topical formulations due to being applied to the thin layers of the skin, so that the gel viscosity plays an important role in controlling of drug permeation.  Figure 1 shows the behavior of carbopol and BG5 that had a viscosity tested at six times with 10, 20, 30, 40, 50, and 60 rpm.

Generally, the viscosity of gel formulations reflects consistency [19]. The viscosity of these gels decreases with increasing rate of shear, showed with non-Newtonian flow (shear thinning); this behavior is preferred due to its low flow resistance when applied at high shear conditions [23]. This viscosity decreases with possible pseudoplastic behavior observed in the formulation of BG5, confirms the characteristic of high spreadability due to the decrease in viscosity when applying certain force, and at the same time has the property of remaining at the application site without drain [24].

## 4. Conclusions

According to the results obtained from this study, it was concluded that borneol was successfully incorporated into the carbopol formulation to obtain a gel. Borneol gel showed good pH value, conductivity, viscosity, spreadability, and stability before and after the 12 days in the freeze-thaw cycle. Therefore, it was concluded that the formulation could be very promising alternative for the topical or transdermal treatment. However, further preclinical, clinical, and long-term stability studies should be required.

## Figures and Tables

**Table 1:** Results of evaluation of the preliminary stability of borneol gel 5% before and after the freeze-thaw cycle.

Sample	Appearance	pH	Conductivity	Centrifugation
BG5% before	Homogeneous, transparent, colorless	3.95 ± 0.01	186.15 ± 0.35 mV	No noticeable instability in the formulation.
BG5% after	Homogeneous, transparent, colorless	3.83 ± 0.03	171.3 ± 1.27 mV	No noticeable instability in the formulation.

Values are mean ± standard deviation.
